# Anatomic distribution, clinical features, and survival data of 87 cases primary gastrointestinal lymphoma

**DOI:** 10.1186/s12957-016-0821-9

**Published:** 2016-03-18

**Authors:** Zheng Ge, Zhong Liu, Xiang Hu

**Affiliations:** Department of General Surgery, The First Affiliated Hospital of Dalian Medical University, Dalian, China; Huaihe Hospital of Henan University, Kaifeng, China

**Keywords:** Primary gastrointestinal lymphoma, Clinical features, Treatment, Prognosis

## Abstract

**Background:**

The purpose of this study is to analyze the anatomic distribution, clinical features, therapeutic methods, and prognosis factors of primary gastrointestinal lymphoma (PGIL).

**Methods:**

Clinical data of 87 cases PGIL in the First Affiliated Hospital of Dalian Medical University from January 1999 to December 2010 were collected. Follow-ups were made according to the clinical feature, pathological pattern, clinical stage, and therapeutic method. Kapan Meier method was used for the survival analysis. Log-rank test was used to perform univariate survival analysis. COX multivariate analysis was carried out to analyze factors of *P* < 0.05 in univariate survival analysis.

**Results:**

The incidence of PGIL significantly increased in patients more than 40 years old (87.4 %). Clinical symptoms of PGIL were indistinguishable from other digestive system diseases, which included abdominal pain or discomfort (72.4 %), lack of appetite (16.3 %), gastrointestinal hemorrhage (14.9 %), and diarrhea (12.8 %). Some patients presented with systemic symptoms or complications, such as weight loss (35.6 %) and digestive tract obstruction (13.8 %). Primary gastric lymphoma (PGL) was the most common, followed by primary intestine lymphoma (PIL). The majority of PGIL were single lesion, which included 40 cases (87 %) PGL and 35 cases (94.5 %) PIL. The most frequent site of PGL was antrum of the stomach (43.5 %), as to PIL, the small intestine (90.2 %) was the most frequent site, especially within 100 cm far away from ileocecal valve. Most of PGIL were derived from B cell (93.1 %). The most common pathological type was mucosa-associated lymphoid tissue (MALT) (67.4 %) in the PGL group and diffuse large B cell lymphoma (DLBCL) (46.3 %) in the PIL group. Surgical treatment had been performed in most of PGIL, which included 32 cases in the PGL group and 38 cases in the PIL group. The 1-year overall survival (OS) and the 3-year OS were 82 and 77 %, respectively. Analysis of single factor affecting prognosis showed that lesion location, sources of cells, and clinical stage were associated with OS. PGL group had better OS than that of PIL group (1-year 89 vs 62 %, 3-year 84 vs 50 %, *P* = 0.03). B cell-originated group had better OS than that of T cell-originated group (1-year 89 vs 36 %, 3-year 85 vs 0 %, *P* = 0.008). Stage I + II group had better OS than that of stage III + IV group (1-year 89 vs 38 %, 3-year 87 vs 0 %, *P* = 0.007). Multivariate analysis showed that clinical stage and sources of cells were the significant independent prognostic factors.

**Conclusions:**

It was more common to find location of PGIL in the stomach than that in the intestine. The most common pathological type was MALT in the PGL and DLBCL in the PIL. The treatment of PGL was focused on chemotherapy. It was noting that since PIL was not only difficult to make confirmed diagnosis but also likely to develop with complications, so it was usually needed surgical excision. Clinical stage and pathological pattern were related to prognosis of PGIL.

## Background

Deriving from lymphoid tissue beneath mucosa of gastrointestinal wall, primary gastrointestinal lymphoma (PGIL) was extranodal non-Hodgkin’s lymphomas (NHL) that account for 24–49 % of NHL [1]. It used to appear in the stomach and small intestines, accounting for 1–4 % of gastroenteric tumor [2]. PGIL was difficult to identify because clinical features of PGIL was indistinguishable from other gastroenteric diseases, especially from gastrointestinal tumor, which attributes to a high misdiagnosis rate in clinical practice. Besides, the treatment methods were quite different from each other. A retrospective analysis was made on 87 PGIL patients from January 1999 to December 2010 in the First Affiliated Hospital of Dalian Medical University; we summarized their clinical features, pathological patterns, treatment methods, and prognosis factors.

## Methods

1. Patients: clinical data of 87 PGIL patients from January 1999 to December 2010 in the First Affiliated Hospital of Dalian Medical University were collected.
2. Diagnosis and stages standards: all patients were diagnosed according to the Dawson standard of gastrointestinal lymphoma, which included (1) absence of perpheral lymphadenopathy at the time of presentation; (2) lack of enlarged mediastinal lymph nodes; (3) normal total and differential white blood cell count; (4) predominance of bowel lesion at the time of laparotomy with only lymph nodes obviously affected in the immediate vicinity; and (5) no lymphomatous involvement of liver and spleen.
  Clinical stages were established according to Ann Arbor staging with Musshoff modification. Stage I: lesions are confined to the gastrointestinal tract, under the side of the diaphragm, without lymph node metastasis. Stage II: lesions invade from the gastrointestinal tract to abdominal cavity, with peritoneal lymph node involvement. Stage III: lesions are confined to the gastrointestinal tract, with lymph node metastasis on both sides of the diaphragm. Stage IV: huge tumor with or without lymph node metastasis, and diffuse non-gastrointestinal tract organs or tissues are involved.
  Pathological diagnosis was referred to the classification of lymphoma by the World Health Organization (WHO). The main pathological patterns of primary gastrointestinal lymphoma are (1) B cell mucosa-associated lymphoid tissue (MALT); (2) diffuse large B cell lymphoma (DLBCL); (3) mantle cell lymphoma (MCL); and (4) enteropathy-associated T cell lymphoma (EATL).
3. Treatment: 87 patients were divided into operation group and non-operation group. Operation group included surgery alone and surgery combined with postoperative chemotherapy or other treatment. All chemotherapy plans were CHOP plan (phosphoric acid amide + doxorubicin + vincristine + prednisone). Rituximab was used in biotherapy.
4. Follow-up: 64 patients finished follow-up visit by phone until December 2013, and the rate of follow-up was 73.6 %. Follow-up information was obtained through follow-up ambulatory visits and phone contacts with patients or their family members. Survival time was measured from the date of diagnosis to death from any cause or to the last follow-up.
5. Statistical approaches: we used SPSS 21.0 software to analyze data. Kapan Meier method was used for the survival analysis. Log-rank test was used to perform univariate survival analysis. COX multivariate analysis was carried out to analyze factors of *P* < 0.05 in univariate survival analysis. *P* < 0.05 was considered to indicate statistically significant.
6. Ethics statement: this study was approved by the Ethical Committee of First Affiliated Hospital of Dalian Medical University, and the reference number was LCKY2013-46.

## Results

1. General information: there were 45 males and 42 females in 87 PGIL patients with the male-to-female ratio of 1.07:1. The average age was 57.3 years old, ranged from 15 to 87 years old. Most of patients were older than 40 years old (*n* = 76, 87.4 %); only 11 patients were younger than 40 years old (12.6 %). Symptoms of PGIL were unspecific. The main gastrointestinal symptoms included abdominal pain or discomfort (*n* = 63, 72.4 %), lack of appetite (*n* = 14, 16.3 %), gastrointestinal hemorrhage (*n* = 13, 14.9 %), and diarrhea (*n* = 11, 12.8 %). Some patients presented with systemic symptoms or complications, such as weight loss (*n* = 31, 35.6 %), digestive tract obstruction (*n* = 12, 13.8 %), and perforation (*n* = 6, 6.8 %).
2. Lesion locations (Table 1): 87 patients included 46 cases primary gastric lymphoma, 37 cases primary small intestinal lymphomas, and 4 cases primary colon lymphoma. The majority of PGIL were single lesion, which included 40 cases (87 %) primary gastric lymphoma (PGL) and 35 cases (94.5 %) PIL. As shown in Table 1, the majority of PGL were located at the antrum of the stomach (*n* = 20, 43.5 %), followed by the body of the stomach (*n* = 16, 34.8 %). The most frequent site of PIL was the small intestine (*n* = 37, 90.2 %), which mostly were located at the ileum (*n* = 26, 70.3 %), especially within 100 cm far away from ileocecal valve (*n* = 25).

**Table 1:** Lesion location distribution of 87 patients with PGIL

Lesion location	Case number	Proportion	(%)
Stomach	46		52.9
Antrum of stomach	20	43.5	
Body of stomach	16	34.8	
Fundus of stomach	3	6.5	
Cardia of stomach	1	2.2	
Multiple locations	6	13.0	
Small intestine	37		42.5
Duodenum	3	8.1	
Jejunum	6	16.2	
Ileum	26	70.3	
Multiple locations	2	5.4	
Colon	4		4.6
Total	87		100
3. Pathologic features and stages (Table 2): all patients were non-Hodgkin’s lymphoma, and most of cases were derived from B cell (*n* = 64). In the PGL group, the most common type was MALT (*n* = 31), followed by DLBCL (*n* = 14). In the PIL group, the most common type was DLBCL (*n* = 19), followed by MALT (*n* = 10), and fewer were EATL (*n* = 6). The patients belonged to stage I E 46 cases, stage II E 31 cases, stage III 4 cases, and stage IV 6 cases.

**Table 2:** Pathological patterns of 87 patients with PGIL

Location	Pathological pattern (case number)			
	MALT	DLBCL	EATL	unclassed
Stomach	31	14	0	1
Intestines	10	19	6	6
4. Treatment (Table 3):

**Table 3:** Treatment plan of 87 patients with PGIL

Group	Location	Treating method	Case number	Proportion (%)
Operation group			70	80.5
	Stomach		32	36.8
		Surgery alone	18	20.9
		Surgery with CHOP	10	11.5
		Surgery with R-CHOP	4	4.6
	Intestines		38	43.7
		Surgery alone	17	19.5
		Surgery with CHOP	17	19.5
		Surgery with R-CHOP	4	4.6
Non-operation group			15	17.2
	Stomach		13	14.9
		CHOP	7	8.0
		R-CHOP	3	3.4
		Radical cure of HP	2	2.3
		Rituximab	1	1.1
	Intestines		2	2.3
		CHOP	2	2.3

**Fig. 1:**
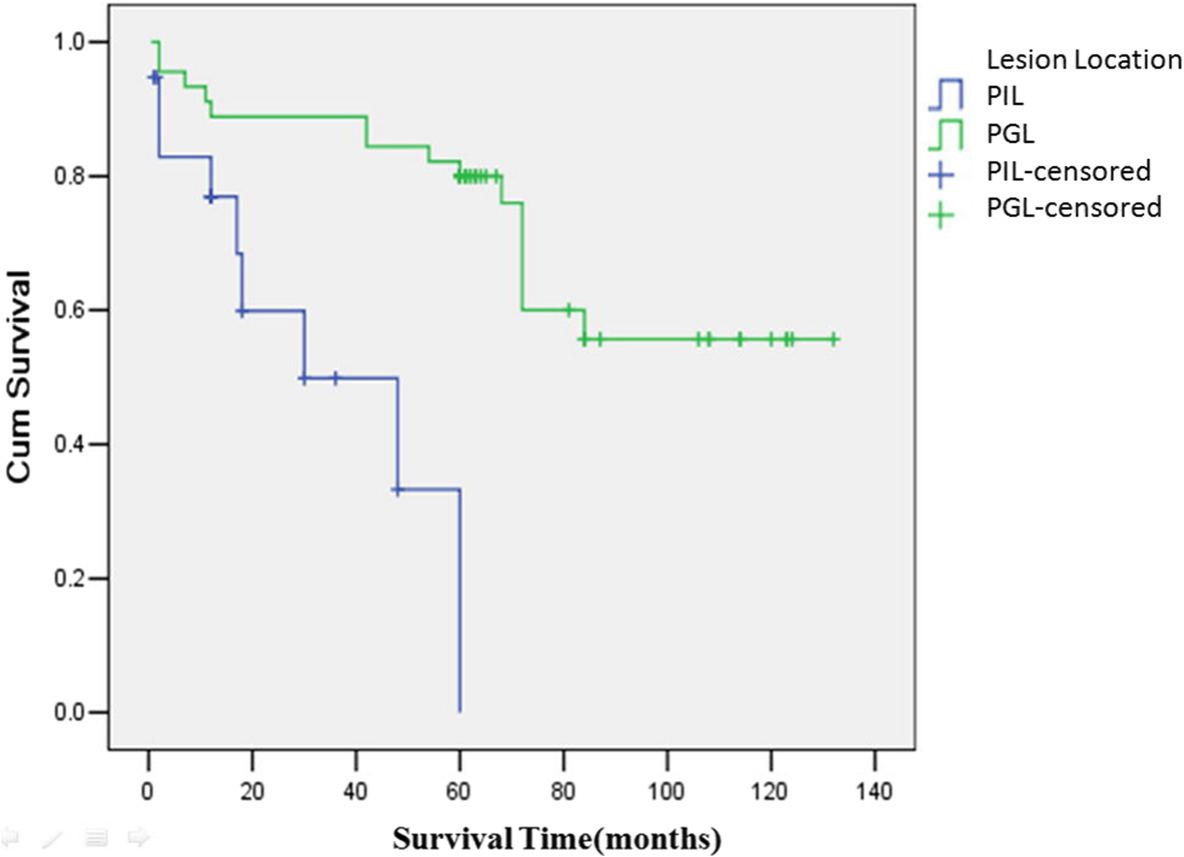
Survival curves of 64 patients with PGIL according to different lesion locations. All 64 patients with PGIL were divided into two groups: PGL group and PIL group, according to different lesion locations. PGL group had better OS than that of PIL group (1-year 89 vs 62 %, 3-year 84 vs 50 %, *P* = 0.03)

**Fig. 2:**
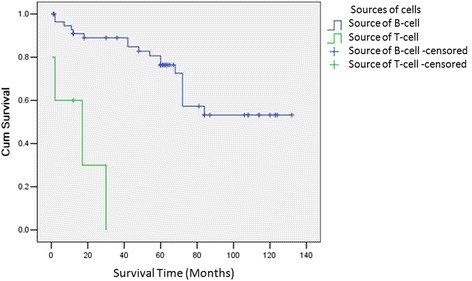
Survival curves of 62 patients with PGIL according to different sources of cells. All 62 patients with PGIL were divided into two groups: source of T cell and source of B cell. B cell-originated group had better OS than that of T cell-originated group (1-year 89 vs 36 %, 3-year 85 vs 0 %, *P* = 0.008)

**Fig. 3:**
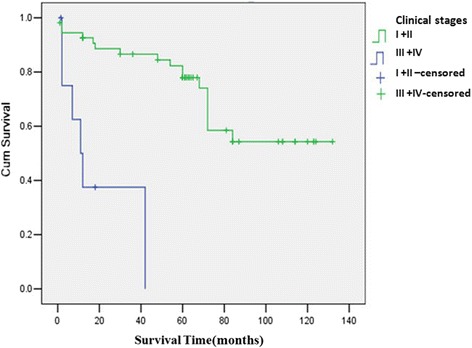
Survival curves of 64 patients with PGIL according to different clinical stages. Stage I + II group had better OS than that of stage III + IV group (1-year 89 vs 38 %, 3-year 87 vs 0 %, *P* = 0.007)

*Two patients abandoned treatment
  As for 46 patients in PGL group, (1) 32 patients accepted surgical treatment, including radical operation (*n* = 27) and palliative surgery (*n* = 5). Radical operation included total gastrectomy (*n* = 6), radical proximal gastrectomy (*n* = 4,) and radical distal gastrectomy (*n* = 17). Eighteen patients accepted surgery alone, and 10 patients were treated with CHOP after surgery, while 4 patients were treated with R-CHOP (rituximab + phosphoric acid amide + doxorubicin + vincristine + prednisone). (2) Thirteen patients with PGL accepted non-operation treatment, including 7 patients treated with CHOP, 3 patients treated with R-CHOP, and 2 patients accepted anti-HP treatment alone. Rituximab alone was used to treat only 1 patient. (3) One patient abandoned treatment after confirmed diagnosis.
  As for 41 patients in PIL group, (1) 38 patients accepted surgical treatment, including 27 patients with radical right hemicolectomy, 16 patients with small bowel resection, and 7 patients with palliative surgery. Seventeen patients accepted surgery alone, and 17 patients were treated with CHOP after surgery, while 4 patients were treated with surgery and rituximab. (2) Two patients accepted non-operation treatment by R-CHOP plan. (3) One patient abandoned treatment after confirmed diagnosis.
5. Follow-up: 64 patients with follow-up information were taken into the survial analysis. The 1-year OS and the 3-year OS were 82 and 77 %, respectively. By univariate analysis, we found that lesion locations, sources of cells, and clinical stage were associated with OS, but surgery did not prolong the survival rate of PGIL compared with other treatments. PGL group had better OS than that of PIL group (1-year 89 vs 62 %, 3-year 84 vs 50 %, *P* = 0.03) (Fig. 1). B cell-originated group had better OS than that of T cell-originated group (1-year 89 vs 36 %, 3-year 85 vs 0 %, *P* = 0.008) (Fig. 2). There was no significant difference of the 1-year and 3-year OS between stage I and stage II, stage III and stage IV patients (data not shown). But stage I + II group had better OS than that of stage III + IV group (1-year 89 vs 38 %, 3-year 87 vs 0 %, P = 0.007) (Fig. 3). Multivariate analysis showed that clinical stage and sources of cells were the significant independent prognostic factors (Table 4).

**Table 4:** Risk factors associated with OS

Item	Case number	Expected average survival time	Univariate analysis			Multivariate analysis	
			OS		*P* value	HR	*P* value
			1 year	3 year			
Gender							
Male	31	79.4	79	75	0.564		
Female	33	89.4	85	79			
Age							
<60 years	37	87.5	83	80	0.216		
≥60 years	27	77.9	80	71			
Location							
Stomach	45	96.5	89	84	0.03	Not significant	
Intestines	19	34.9	62	50			
Size							
<5 cm	34	97.3	82	79	0.142		
≥5 cm	30	70.8	82	74			
Clinical stages							
I + II	55	95.5	89	87	0.003	2.576 (1.024 3.809)	0.001
III + IV	9	20.0	38	0			
Source of cells							
Source of B cells	57	94.7	89	85	0.006	3.119 (1.524 6.384)	0.002
MALT	35	100.9	94	91			
DLBCL	22	85.0	81	74			
Source of T cells	5	14.6	36	0			
EATL	5	14.6	36	0			
Treatment methods							
Surgery alone	22	80.9	81	72	0.604		
Surgery combined other treatment	27	90.5	88	83			
Non-operation	15	49.1	73	73

## Discussion

Although PGIL pathogenesis remained unknown, certain factors had been considered to be related with its incidence for a long time, including virus infection, autoimmune deficiency, and environment pollution. [3, 4]. The incidence of PGIL had increased in Asia, North America, and Europe [5–8]. Many studies showed that the stomach was the most commonly involved site followed by the intestine, while in Pacific Ocean, small intestines were mostly seen followed by the stomach and colon [9, 10]. In our study, we found that 53.9 % patients were PGL and 46.1 % were PIL. Furthermore, the majority of PGL were located at the antrum of the stomach, followed by the body of the stomach, and the most frequent site of PIL was the small intestine, especially within 100 cm far away from ileocecal valve. Besides, it should be noticed in clinical work that there might be multiple lesion locations in PGIL.

Clinical symptoms of PGIL were indistinguishable from other digestive system diseases. The main symptom included abdominal pain or discomfort, together with weight loss and nausea and other intestinal symptoms [11, 12]. Imageological examination might show wall thickened and intestinal masses; it was usually difficult to identify from other gastrointestinal cancer. Endoscopy and biopsy were the most reliable methods for confirming diagnosis [13, 14].

The surgical treatment was traditionally considered as the main treatment methods of PGIL. Most of patients accepted the radical resection. Palliative resection might due to huge size of tumor or extensive transfer of lymph node. However, as lymphoma was highly sensitive to chemotherapy, the main treatment of PGIL was non-surgery now. A prospective study showed that surgery treatment could not improve the 10-year survival rate of PGIL by comparing of surgery plus chemotherapy with chemotherapy alone [6]. Recently, there was a study showed that it had equivalent efficacy whether patients accepted operation or not [11]. Moreover, more and more studies demonstrated that non-surgery strategies had better OS [15, 16]. In our study, 50 patients accepted non-surgery methods, such as CHOP or R-CHOP, which account for 54.5 % of total patients. Rituximab is a chimeric monoclonal antibody against the protein CD20, which is primarily found on the surface of immune system B cells. Rituximab destroys both normal and malignant B cells that have CD20 on their surfaces. The addition of rituximab has improved the overall survival of lymphoma. Many studies have showed that rituximab can improve the efficacy of chemotherapy after relapse [17, 18]. In our retrospective study, there were not all patients whose CD20-positive approved rituximab therapy due to economic reasons. Nowadays, surgery had gradually been replaced by non-surgery treatment. However, many studies showed that surgery was benefit to patients who present with hemorrhage, perforation, or ileus [19, 20], especially to PIL patients. PIL was not only difficult to make confirmed diagnosis but also likely to develop with complications, so it was usually needed surgical excision and then diagnosed by pathologic analysis. In our study, there were 41 PIL patients, which 38 patients accepted surgical treatment. We believed that surgery was the main treatment method of undetermined diagnosis of PGIL patients, with its irreplaceable advantages as follows: (1) surgery was an important means to gain the pathological diagnosis and determine diagnosis; (2) postoperative specimens could be graded and staged correctly in order to judge prognosis more exactly; (3) surgery might alleviate tumor load, relieve clinical symptoms, and strengthen effect of other treatment, such as postoperative chemotherapy; and (4) patients who were insensitive to other treatment or appeared life-threatening complications should choose surgery as soon as possible.

In summary, the reasonable clinical treatment method of PGIL should be made according to the location, clinical stage, pathologic pattern, and with complications or not.

## Conclusions

In conclusion, our study showed that it was more common to find location of PGIL in the stomach than that in the intestine. The most common pathological type was MALT in the PGL group and DLBCL in the PIL group. The treatment of PGL was focused on chemotherapy. It was noting that since PIL was not only difficult to make confirmed diagnosis but also likely to develop with complications, so it was usually needed surgical excision and then diagnosed by pathologic analysis. Clinical stage and pathological pattern were related to prognosis of PGIL.

### Consent

Written informed consent was obtained from the patient for the publication of this report and any accompanying images.
